# Assessment of Biochemical Composition of Fruits of *Hippophae rhamnoides* (Elaeagnaceae juss.), *Viburnum opulus* (Viburnaceae raf.) and *Lonicera caerulea* subsp. *altaica* (Caprifoliaceae juss.)

**DOI:** 10.3390/metabo15040256

**Published:** 2025-04-09

**Authors:** Tatiana Vdovina, Olga Lagus, Andrei Vinokurov, Zhanar Aimenova, Aidar Sumbembayev

**Affiliations:** Altai Botanical Garden, Ridder 070000, Kazakhstan; tvdovina2017@mail.ru (T.V.); lelik_ridder1994@mail.ru (O.L.); anvin64@mail.ru (A.V.); aimenova.zhanara@mail.ru (Z.A.)

**Keywords:** Altai Mountain region, vitamins, sugar content, phenolic compounds, carotenoids, flavonoid composition, HPLC

## Abstract

**Background/Objectives:** This study explores the biochemical diversity of *Hippophae rhamnoides*, *Viburnum opulus*, and *Lonicera caerulea* subsp. *altaica* to identify genotypes rich in bioactive compounds for breeding nutritionally valuable cultivars. **Methods:** Selected forms and cultivars of each species were evaluated for key biochemical traits. Analyses included quantification of vitamins (C, B9, B12), sugars, organic acids, carotenoids, and flavonoids using HPLC and TLC. **Results:** In *H. rhamnoides*, vitamin C content ranged widely, with ‘Pamyati Baytulina Sh-9-81’ reaching 156.0 mg/%, while ‘Shetlastinka No. 7’ showed the most favorable sugar-to-vitamin C ratio. ‘Krasnoplodnaya K-14-81’ had the highest carotenoids (55.3 mg/100 g), and ‘Dolgozhdannaya No. 5’ was notable for flavonoid richness. In *V. opulus*, considerable variation was observed in vitamin content, sugars, and dry matter; ‘Zhemchuzhnoe Ozhele’ and ‘Shtambovaya’ were rich in rutin and robinin. In *L. caerulea* subsp. *altaica*, forms No. 5, 7, and 9 stood out for vitamin C, sugar, and flavonoid content. Hyperoside, isorhamnetin, and myricetin were common, while kaempferol and hypolaetin were form-specific. **Conclusions:** *H. rhamnoides* demonstrated the highest variability in biochemical composition, while *L. caerulea* subsp. *altaica* showed a particularly rich flavonoid profile. These species offer valuable genetic resources for developing biofortified cultivars suited to both nutritional and adaptive breeding goals.

## 1. Introduction

The modern research strategy in the study of fruit and berry plants within the fields of botany, genetics, biotechnology, breeding, and phytochemistry is focused on their introduction into cultivation, with an emphasis on productivity, fruit quality, ornamental value, and adaptability to environmental conditions [1,2,3,4,5,6,7,8,9,10]. Among the wild species of fruit and berry plants growing in the East Kazakhstan region, *Hippophae rhamnoides* L., *Viburnum opulus* L., and *Lonicera caerulea* subsp. *altaica* (Pall.) Gladkova have been the most extensively studied in the Altai Botanical Garden (ABG), both in their natural habitats and under cultivation. Long-term research has demonstrated their advantages, including high winter hardiness, productivity, adaptability, and valuable fruit properties. Additionally, global experience in the introduction of these species serves as an important factor in the conservation of plant biodiversity [11,12,13,14,15].

The biochemical composition of fruits is a critical aspect of breeding programs for fruit and berry crops, as they represent the most valuable part of the plant and have long been used as food and in traditional medicine. The increasing interest in the chemical composition of *H. rhamnoides*, *V. opulus*, and *L. caerulea* subsp. *altaica* is attributed to their rich content of biologically active substances (BAS), including ascorbic acid (vitamin C), phenolic compounds, bioflavonoids, flavonoids, and carotenoids, which are known for their antioxidant properties. These compounds can enhance the resistance of organisms to environmental pollution and stress factors [16,17,18,19,20].

Medicinal plants containing flavonoids, such as rutin, robinin, myricetin, kaempferol, hypolaetin, isorhamnetin, and hyperoside, are considered promising sources for herbal medicine production [21,22,23]. Due to their widespread distribution and structural diversity, these flavonoids have become a focal point of research in pharmacognosy, pharmacy, and medicine [24,25,26]. Their antioxidant, anti-inflammatory, anti-mutagenic, and anti-carcinogenic properties, as well as their ability to modulate key cellular enzymes, make them valuable bioactive compounds. Furthermore, numerous studies have demonstrated the potential of flavonoids in mitigating neurodegenerative diseases due to their antioxidant and anti-inflammatory effects [27]. The incorporation of wild fruits and berries rich in BAS (e.g., vitamins C and PP, carotenoids, and flavonoids) into functional food and nutraceutical products could significantly expand the range of eco-friendly food products [23,28,29].

A crucial direction in the breeding of fruit and berry plants involves the study of the biochemical composition of fruits, utilizing genetic resources from various countries, including Korea [30], Iran [31], Lithuania [32], Russia [33,34], and Belarus [35]. ABG actively studies, preserves, and expands the genetic pool of fruit and berry plants, with ongoing successful breeding work on *H. rhamnoides* [36]. The breeding and genetic fund of this species at ABG consists of 7 developed varieties (Yubileinaya Kotukhova, Shetlastinka, Pamyati Baytulina, Feyerverk, Fakel, Plakuchaya, and Asem), 45 forms, 25 families, and 53 promising seedlings. The introduction population of *V. opulus* includes 28 formo-clones and 71 plants from three coenopopulations: Barkhotinskaya, Cheremshanskaya, and Ulbastroevskaya [36]. *L. caerulea* subsp. *altaica* is represented by 22 forms, 105 seedlings of elite forms (Golubaya Volna and No. 2), and 26 clones of the Golubaya Volna form. The introduction of selected forms of these species into cultivation highlights their potential for widespread cultivation in Kazakhstan and their suitability as genetic resources for breeding.

Our research focuses on studying the biological characteristics of selected wild berry forms under cultivation and analyzing their content of BAS, including vitamins C, B2, B9, and B12, total sugar content, acidity, soluble solids, carotenoids, phenolic compounds, and flavonoids. While previous studies have examined the medicinal and nutritional potential of these species, this research fills a gap in the detailed biochemical profiling of multiple varieties and forms within these fruit-bearing plants. The study was aimed to facilitate the use of these species as sources of valuable traits in breeding programs. The primary objective of this study is to investigate the variability of the chemical composition of fruits in *H. rhamnoides*, *V. opulus*, and *L. caerulea* subsp. *altaica* and to identify varieties and forms with optimal levels of BAS and flavonoids. This research aims to provide new source material for the breeding of these fruit and berry crops, with an emphasis on evaluating genotypic diversity and the variation of biochemical parameters in the studied species.

## 2. Materials and Methods

### 2.1. Collection of Material

**Figure 1:**
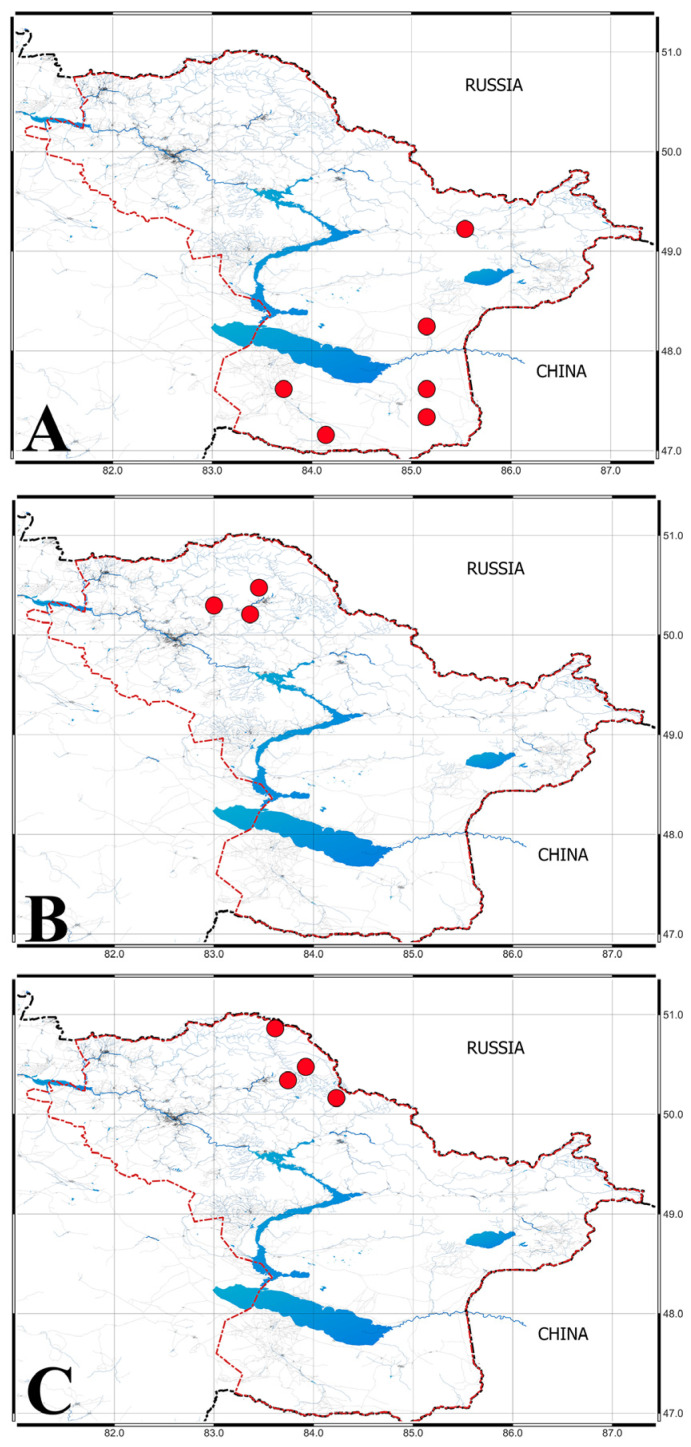
Sampling points of the fruits of the studied plants. (**A**)—*H. rhamnoides*, (**B**)—*V. opulus*, (**C**)—*L. caerulea* subsp. *altaica*. Red dots denote collection points, blue area are lakes and rivers. Black line denotes border of Kazakhstan, red line denotes border of East Kazakhstan region.

Fruits of 16 varieties and cultural forms (breeding lines) of *H. rhamnoides* and 5 varieties of *V. opulus* were taken from ABG collection. Other fruit samples of *V. opulus* and all samples of *L. caerulea* subsp. *altaica* were collected from natural populations in Altai Mountains. Fruits were collected from natural habitats in the Kazakh part of the Altai Mountain region (Figure 1). The plots where the fruits of *V. opulus* and *L. caerulea* subsp. *altaica* were collected located on the southwestern slope of the mountain Belkina. The soils were chernozem-like silty loams (humus content 6.4%). For *H. rhamnoides*, the collection was from 25 August to 5 September 2024. The age of the plants was 17–19 years. For *V. opulus*, the collection was at the end of September, 2024. The age of the plants was 12–14 years. The collection of *L. caerulea* subsp. *altaica* fruits was carried out in the third ten-day period of June, 2024. The age of the plants was 18–20 years. Fruits were harvested at full ripeness and subsequently dried in an oven at 105 °C to ensure uniform moisture content before biochemical analysis.

### 2.2. Assessment of Biochemical Composition

The biochemical composition of fruits from *H. rhamnoides*, *V. opulus*, and *L. caerulea* subsp. *altaica* was assessed based on various parameters representing different classes of bioactive compounds. Total sugars were quantified using refractometry. Dried fruits were homogenized and the juice was extracted by centrifugation at 6000 rpm for 10 min. After the calibration with distilled water, the refractive index is measured, and the total soluble solids (TSSs) are recorded in °Bx. Total sugar content (%) was estimated using the formula: Total Sugars (%) = TSSs(°Bx) × F, where F was factor accounting for the presence of non-sugar solubles. Organic acids were identified using thin-layer chromatography (TLC) on 10 × 20 cm TLC plates in an acetone–water–chloroform–ethanol–ammonium hydroxide (60:2:6:10:22) solvent system.

Carotenoid content was analyzed using high-performance liquid chromatography (HPLC) LC-20 (Shimadzu, Kyoto, Japan). All-trans-utein (X6250-1MG) and zeaxanthin (14681-1MG-F) were purchased from Sigma-Aldrich (St. Louis, MO, USA) and used as standards. For the analysis, 0.5 g of dried fruits was homogenized in 10 ML of cold acetone and centrifuged at 5000 rpm for 10 min. The extract was dried under vacuum and reconstituted in 1 mL of mobile phase (methanol/acetonitrile/ethyl acetate, 50:25:25, *v*/*v*/*v*). For the analysis, C18 (250 × 4.6 mm, 5 μm) column was used. The following parameters were used: flow rate of 1.0 mL/min, mobile phase (methanol/acetonitrile/ethyl acetate, 50:25:25, *v*/*v*/*v*), and injection volume of 20 μL. The signal was detected at 450 nm using SPD-20A.

Tocopherols were measured using an LC-20 liquid chromatograph (Shimadzu) equipped with an SPD-20A spectrophotometric detector. For the analysis, 0.5 g of dried fruits was homogenized in a 10 ml mixture of methanol/acetonitrile (99:1, *v*/*v*) and centrifuged at 5000 rpm for 10 min. The extract was dried under vacuum and reconstitute in 1 mL of mobile phase (methanol/acetonitrile, 99:1, *v*/*v*). Solutions were filtered through a 0.45 μm membrane filter, and the chromatographic separation was performed at a constant flow rate of 1 ml/min. For the analysis, C18 (250 × 4.6 mm, 5 μm) column was used. Detection was carried out at 292 nm UV wavelength, with the column maintained at 30 °C. A freshly prepared α-tocopherol solution in ethanol (0.2 mg/ml) served as the standard. The standard curve was generated by plotting the concentration (mg/ml) against the peak area of α-tocopherol. Sigma-Aldrich (St. Louis, MO, USA) α-tocopherol was used as the reference standard.

Vitamins B2, B9, B12, PP, and C were quantified using the LC-20 liquid chromatograph (Shimadzu) with C18 (250 × 4.6 mm, 5 μm) column and an SPD-20A spectrophotometric detector set at 270 nm. Standards of these vitamins were purchased from Sigma-Aldrich (St. Louis, MO, USA). The preparation of the sample included extraction from 1 g of dried fruits with 10 mL of 0.1% HCl (for vitamins B2, B9, B12, and PP) or 5% metaphosphoric acid (for vitamin C), sonication for 30 min, centrifugation at 10,000 rpm for 10 min, and filtration through a 0.22 µm membrane filter before HPLC analysis. For vitamins B2, B9, B12, and PP, mobile phase included solvent A (0.1% trifluoroacetic acid) and solvent B (acetonitrile) with gradient elution of 5–30% B over 30 min. For vitamin C, isocratic elution was performed with 0.1% formic acid/methanol (95:5). Detection was performed at 450 nm for B2, at 280 nm for B9, at 260 nm for B12, and at 245 nm for vitamin C.

The flavonoid composition of fruits was analyzed using LC-20 in isocratic elution mode with a diode array detector. For the analysis, 0.5 g of dried fruits was mixed with 10 mL of 70% methanol, sonicated for 30 min and centrifuged at 10,000 rpm for 10 min. Next, the supernatant was filtered through a 0.22 µm membrane filter. The mobile phase included solvent A (0.1% formic acid) and solvent B (acetonitrile). The analysis was performed in gradient elution: 0–10 min—20–40% B; 10–20 min—40–60% B; 20–30 min—60–80% B; 30–35 min—80–20% B (re-equilibration). Separation was performed at a constant flow rate of 1 mL/min at 30 °C using C18 (250 × 4.6 mm, 5 μm) column. Spectral data were recorded using diode array in the wavelength range of 200–400 nm. All standards of flavonoids were purchased from Sigma-Aldrich (St. Louis, MO, USA).

A total of 16 forms of *H. rhamnoides* and *L. caerulea* subsp. *altaica*, along with 15 forms of *V. opulus*, were analyzed for BAS. Additionally, flavonoid composition was examined in 7 forms of *H. rhamnoides*, 13 forms of *V. opulus*, and 10 forms of *L. caerulea* subsp. *altaica.*

### 2.3. Statistics

Given the genetic diversity of the studied species, the data were grouped into interval classes based on the concentration of each chemical compound. For vitamin C, carotenoids, and phenolic substances, values were classified into three categories: low, medium, and high. For total sugar content, titratable acidity, and soluble solids—where the variability range was narrower—data were categorized into two groups: low and high. This classification allowed for the assessment of each form and variety as a potential natural source of bioactive compounds under the specific environmental conditions studied. Based on the degree of variability, chemical substances were classified into four groups: low variability (coefficient of variation (C%) ≤ 12%), medium variability (12% < C% ≤ 20%), high variability (20% < C% ≤ 40%), and very high variability (C% > 40%). The study employed biometric methods based on statistical and probabilistic patterns. The obtained data were processed using analysis of variance (ANOVA) [37], considering parameters such as standard error of the mean, tabulated t-test values at a 0.05 significance level, C%, and experimental accuracy (P%). Statistical analysis was performed using the R statistics software packages [38].

## 3. Results

### 3.1. Biochemical Composition of Varieties and Forms of H. rhamnoides

As part of the research on BAS, 16 forms of *H. rhamnoides* were analyzed, and the flavonoid composition was assessed in seven varieties.

Among natural vitamins, vitamin C was of particular significance. Biochemical studies demonstrated that its content in the fruits of the selected *H. rhamnoides* forms and varieties varied considerably, ranging from 23.40 mg/% in the Yubileynaya Kotukhova (2-22) III variety to 156.0 mg/% in the Pamyati Baytulina Sh-9-81 (4-6) II variety. The average content was 66.64 mg/% (Table 1).

The highest content of vitamin C was observed in the following varieties and forms: Pamyati Baytulina Sh-9-81 (4-6) II—156.00 mg/%, Dolgozhdannaya No. 5 (3-24) III—146.64 mg/%, Solnyshko (1-18) III—140.92 mg/%, and No. 7 (2-24) III—120.38 mg/% (Table 1).

The soluble sugar content in the studied *H. rhamnoides* forms varied between 3.45% and 4.35%. The following sweet-fruited forms were distinguished: Vitaminka (3-20) II, Kan-2-86 (2-4), and the varieties Fakel K-14-81 (3-25) III, Feyerverk K-14-81 (3-4), and Shetlastinka No. 7 (2-24) III. The most favorable combination of sugars and vitamin C was observed in the varieties Fakel K-14-81 (3-25) III (4.13% sugar, 57.20 mg/% vitamin C) and Shetlastinka No. 7 (2-24) III (4.35% sugar, 120.38 mg/% vitamin C).

The organic acid content in *H. rhamnoides* fruits ranged from 1.35% to 2.86%. The highest acidity was recorded in the forms Solnyshko (2.86%), Krasnoplodnaya K-14-81 (2.79%), and Lyubimaya (2.58%). The lowest acidity (1.35%) was characteristic of the Vitaminka T-14-86 (3-3) form, which had a positive effect on taste.

The dry matter content in the fruits varied from 8.2% to 11.8%, with the highest accumulation observed in the Sh-9-81 (3-12) III form (11.8%) and the lowest in the Plakuchaya T-2-82 (2-32) III variety (8.2%).

For the analysis of carotenoids, 16 promising forms of *H. rhamnoides* were selected, nine of which exhibited a reddish-orange fruit color. Biochemical analyses revealed a significant variation in carotenoid content, ranging from 8.39 mg/100 g in the Shetlastinka No. 7 (2-24) III variety to 55.3 mg/100 g in the Krasnoplodnaya K-14-81 (4-27) III form (Table 1). High levels of carotenoids were recorded in the following forms and varieties: Vitaminka (3-20)—39.13 mg/100 g, Pamyati Baytulina Sh-9-81 (4-6) II—25.04 mg/100 g, and Yantarnaya (2-1)—24.17 mg/100 g. Most forms (56.2%) were characterized by a moderate accumulation of carotenoids (20–40 mg/100 g). The highest carotenoid accumulation was observed in varieties Krasnoplodnaya K-14-81 (4-27) III (55.30 mg/100 g), Vitaminka T-14-86 (3-20) III (39.13 mg/100 g), and Fakel K-14-81(3-17) III (Fakel K-14-81(3-17) III).

The fruits of *H. rhamnoides* were found to be rich in biologically active phenolic compounds, including catechins, anthocyanins, flavanones, and flavonols. Their content ranged from 41.9 to 105.8 mg/100 g (Table 1). The highest levels of phenolic substances were recorded in the varieties Pamyati Baytulina Sh-9-81 (4-6) II (105.8 mg/100 g), Solnyshko (1-18) III (100.46 mg/100 g), Dolgozhdannaya No. 5 (3-24) III (92.0 mg/100 g), and Lyubimaya T-2-82 (3-14) II (87.7 mg/100 g).

Seven promising varieties and forms of *H. rhamnoides* (fruit size and high content of BAS) were selected for the flavonoid composition analysis. Five flavonoids (robinin, rutin, gallic acid, hypolaetin, and hyperoside) were identified in one variety, Dolgozhdannaya No. 5 (3-24) III, and only three compounds (robinin, rutin, and gallic acid) were identified in the remaining six varieties and forms (Table 2).

In terms of the quantitative composition of flavonoids in the fruits of *H. rhamnoides*, robinin and rutin were the most prominent compounds. Gallic acid was present in insignificant quantities, while hypolaetin and hyperoside were detected in trace amounts only in the form Dolgozhdannaya No. 5 (3-24) III (Table 2).

Robinin content varied among the studied *H. rhamnoides* forms, ranging from 1.31 mg/100 g in the form Vitaminka T-14-86 (3-20) III to 7.52 mg/100 g in the variety Asem Sh-9-81 (3-27) III (Table 2). The average value for robinin across the studied forms was 4.18 mg/100 g, with a coefficient of variation of 32.12%. The Dolgozhdannaya No. 5 (3-24) III variety exhibited a high robinin content (4.80 mg/100 g), as did the Shetlastinka No. 7 (2-24) III (4.72 mg/100 g) and Fakel K-14-81 (3-17) III (4.52 mg/100 g). The remaining forms demonstrated lower values.

Rutin content ranged from 1.19 mg/100 g in the Yubileynaya Kotukhova T-2-82 (2-22) III variety to 4.76 mg/100 g in the Fakel K-14-81 (3-17) III variety, with an average value of 2.67 mg/100 g (Table 2). A high accumulation of rutin was observed in two forms: Krasnoplodnaya K-14-81 (4-27) III (3.69 mg/100 g) and Vitaminka T-14-86 (3-20) III (2.96 mg/100 g), as well as in the variety Fakel K-14-81 (3-17) III (4.76 mg/100 g).

Gallic acid was present in trace amounts, ranging from 0.21 mg/100 g in the Yubileynaya Kotukhova T-2-82 (2-22) III variety to 0.72 mg/100 g in the Dolgozhdannaya No. 5 (3-24) III form (Table 2). The average content of gallic acid was 0.35 mg/100 g. Despite this relatively low content, the coefficient of variation was high (41.0%). The highest accumulation of gallic acid was recorded in two forms: Dolgozhdannaya No. 5 (3-24) III (0.72 mg/100 g) and Krasnoplodnaya K-14-81 (4-27) III (0.41 mg/100 g).

The flavonoids hypolaetin and hyperoside were detected in small amounts only in the form Dolgozhdannaya No. 5 (3-24) III, at concentrations of 0.18 mg/100 g and 1.28 mg/100 g, respectively.

### 3.2. Varieties and Forms of H. rhamnoides Distinguished by Their Biochemical Composition

Seven varieties and forms of *H. rhamnoides* exhibit distinct biochemical profiles, characterized by variations in fruit morphology, productivity, and the accumulation of key bioactive compounds.

**Figure 2:**
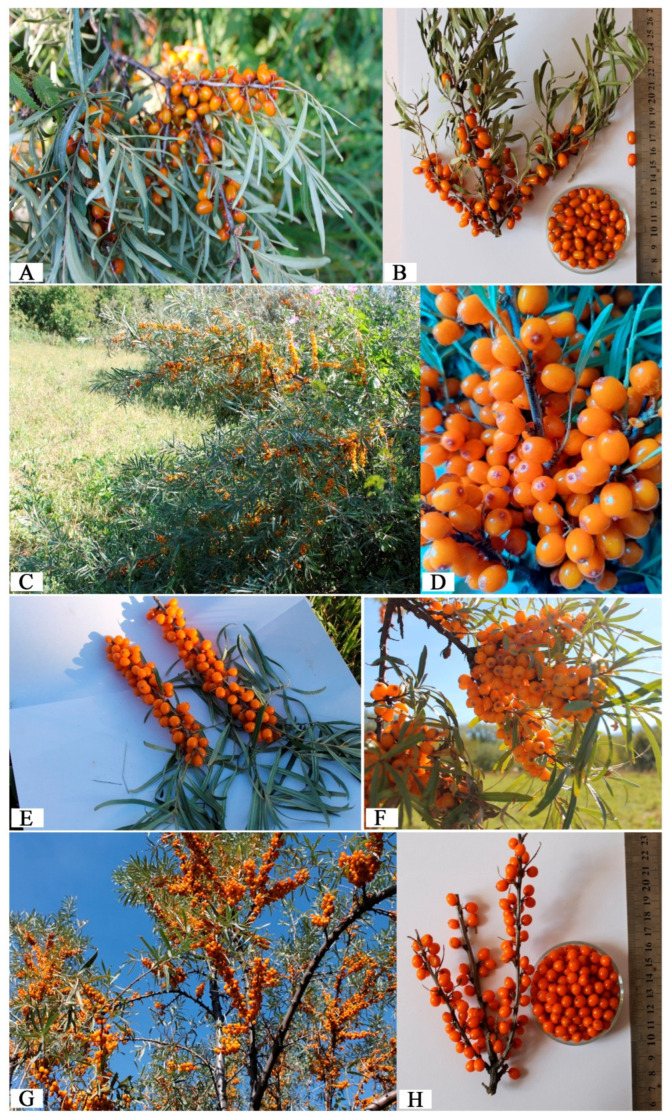
Distinguished varieties and forms of *H. rhamnoides*. (**A**)—Variety Pamyati Baytulina; (**B**)—Fruits of the variety Pamyati Baytulina; (**C**)—Form Solnyshko (1-18) III; (**D**)—Fruits of the form Solnyshko (1-18) III; (**E**)—Variety Shetlastinka; (**F**)—Form Dolgozhdannaya No. 5 (3-24); (**G**)—Variety Fakel K-14-81 (3-17) III; (**H**)—Fruits of the variety Fakel K-14-81 (3-17) III.

Pamyati Baytulina (Figure 2A,B) is a compact, large-fruited variety with a height of 3.9 m and a trunk diameter of 4.6 cm. It has a high productivity of 6.7 kg per bush. The fruits are reddish-orange, cylindrical in shape (9.3 × 7.2 mm), and weigh approximately 55.2 g per 100 fruits. They have a short peduncle (4–5 mm) and a dense arrangement of 24.1 fruits per 10 cm of branch. This variety stands out for its high vitamin C content (156.0 mg/%), dry matter (10.4%), and phenolic compounds (105.8 mg/kg).

Solnyshko (1-18) III (Figure 2C,D) exhibited a mid-season ripening period (first ten days of September). The plants reached a height of 3.9 m, with a stem diameter of 4.7 cm. The fruits were yellow, measuring 9.0 × 7.5 mm, and were easily detachable, requiring a tearing effort of 110 g. The weight of 100 fruits was 52.3 g, and the yield was recorded at 7.8 kg per bush. This variety was a valuable source of vitamin C (120.38 mg/%), carotenoids (8.39 mg/100 g), and phenolic substances (77.5 mg/kg).

Shetlastinka No. 7 (2-24) III (Figure 2E) had a mid-late ripening period. The plants grew to a height of 4.3 m, with a stem diameter of 5.0 cm. The fruits were large, barrel-shaped, and orange, measuring 9.4 × 6.9 mm, with a weight of 100 fruits recorded at 68.2 g. The yield reached 11.0 kg per bush. This variety demonstrated high winter hardiness and was notable for its high vitamin C content (120.3 mg/%) and phenolic substances (77.5 mg/100 g).

Dolgozhdannaya No. 5 (3-24) III (Figure 2F) had an average ripening period. The plants grew to 4.2 m in height, with a stem diameter of 4.9 cm. The fruits were large, yellowish-orange, and barrel-shaped, measuring 10.0 × 6.8 mm, with a weight of 100 fruits at 72.8 g. The yield was 9.7 kg per bush. This variety had the highest recorded vitamin C content (146.6 mg/%) and phenolic substances (92.0 mg/kg). It was identified as a promising donor of five flavonoids: robinin (4.80 mg/100 g), rutin (1.63 mg/100 g), hyperoside (1.28 mg/100 g), gallic acid (0.72 mg/100 g), and hypolaetin (0.18 mg/100 g).

Fakel K-14-81 (3-17) III (Figure 2G,H) exhibited a mid-late ripening period, beginning in the second ten days of September. The plants reached a height of 4.8 m, with a stem diameter of 4.2 cm. This variety was characterized by the upward growth direction of the “cobs”. The cylindrical fruits measured 10.4 × 7.5 mm, with a weight of 100 fruits at 46.1 g. The fruit density on the “cob” was 30 fruits per 10 cm. The yield was recorded at 5.7 kg per bush. This variety accumulated significant amounts of carotene (31.4 mg/100 g) and vitamin C (57.2 mg/%). It was also valuable for its robinin content (4.52 mg/100 g) and rutin (4.76 mg/100 g).

In terms of composition, three forms were distinguished by a high rutin content: Dolgozhdannaya No. 5 (3-24), Krasnoplodnaya K-14-81 (4-27) III, and Vitaminka T-14-86 (3-20) III, along with one variety, Fakel K-14-81 (3-17) III. A relatively high concentration of robinin was observed in two varieties, Shetlastinka No. 7 (2-24) III and Fakel K-14-81 (3-17) III, as well as in one form, Dolgozhdannaya No. 5 (3-24). A significant accumulation of gallic acid was recorded in the forms Dolgozhdannaya No. 5 (3-24) and Krasnoplodnaya K-14-81 (4-27) III.

### 3.3. Biochemical Composition of Varieties and Forms of V. opulus

The introduced population of *V. opulus*, consisting of 28 formo-clones and 71 plants, was studied. The biochemical composition of 15 forms and the flavonoid content of 13 forms were analyzed. To develop adaptive varieties, it was essential to investigate the biochemical composition of the fruits. The vitamin C content ranged from 116.18 mg/% to 346.85 mg/% and was classified into three groups: low (116.1–193.0 mg/100 g), medium (194.0–269.9 mg/100 g), and high (269.0–346.0 mg/100 g). The average vitamin C content was 185.42 mg/%, with a variation coefficient of 33.44% (Table 3).

A significant accumulation of vitamin B9 (folic acid) was observed in the forms Shtambovaya K (45-1) (193.24 mg/100 g) and K (35-4) (194.16 mg/100 g). Its variation ranged from 64.96 mg/100 g in the form K (45-2) to 193.24 mg/100 g in the form Shtambovaya K (45-1). The average value was 111.00 mg/100 g, with a variation coefficient of 34.75%. A low content of this vitamin (64.9–107.9 mg/100 g) was noted in 40.0% of the forms, while an average content (108.0–150.9 mg/100 g) was found in 46.6% of the forms.

The highest vitamin B12 content was observed in the forms Shtambovaya (45-1) (11.52 mg/100 g) and Businka (24-7) (10.72 mg/100 g), identifying them as potential donors of this vitamin. The variation in vitamin B12 content ranged from 5.06 mg/100 g to 11.52 mg/100 g, with an average value of 7.99 mg/100 g and a variation coefficient of 23.58%. The distribution of forms was nearly uniform, with seven forms exhibiting high values (>8.32 mg/100 g) and eight forms showing low values (<8.31 mg/100 g).

No significant differences were found in the accumulation of vitamin B2, with its content varying from 0.15 to 1.6 mg/100 g. The average value was 1.21 mg/100 g. The distribution of forms was divided into two groups: those with high values (>1.37 mg/100 g) (six forms) and those with low values (<1.36 mg/100 g) (nine forms).

During the selection of *V. opulus* forms, particular attention was given to identifying those with a reduced titratable acid content (<2.25%) and an increased soluble sugar content (>8.5%). Acidity exhibited moderate variability, with a variation coefficient of 13.48%. It ranged from 1.76% to 2.77%, with an average value of 2.16%. Nine forms with an acidity level of 1.76–2.26% were considered promising for further breeding.

The sugar content varied slightly among the forms, ranging from 7.40% to 9.6%, with an average value of 8.71%. This trait exhibited low variability, with a variation coefficient of 8.71%. An increased total sugar content (>8.5%) was recorded in six forms: Blestyashchaya (45-7), Shtambovaya (45-1), K (27-4), Nezhenka (42-1), K (23-6), and K (23-8).

The SAI plays an important role in taste improvement. High SAI values were observed in five forms: Blestyashchaya (45-7), K (27-4), Nezhenka (42-1), K (23-6), and K (27-5). The average value for SAI was 3.92%, with moderate variability (variation coefficient: 13.38%).

Dry matter content exhibited slight variations, ranging from 13.7% in the form K (22-5) to 18.0% in the form Nezhenka (42-1). The average value was 15.45%, with a variation coefficient of 8.85%, indicating low variability. An increased dry matter content (>16.2%) was observed in the forms Blestyashchaya (45-7), Shtambovaya (45-1), K (27-4), Nezhenka (42-1), K (23-6), and K (23-8).

**Figure 3:**
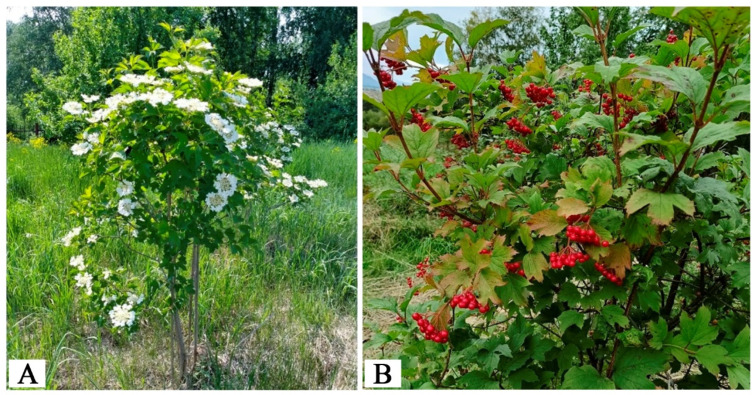
*V. opulus* form Shtambovaya (45-1). (**A**)—Flowering. (**B**)—Fruiting.

The *V. opulus* form Shtambovaya (45-1) (Figure 3A) exhibited a combination of valuable agronomic traits and a high content of BAS. The plant reached a height of 1.3 m, with a trunk length of 0.6 m and a stem diameter of 2.1 cm. It demonstrated winter hardiness and resistance to pests and diseases. The brush size measured 6.28 × 6.86 cm, with 107 flowers and 39.5 fruits per brush (Figure 3B). The fruits were medium-sized (100 pcs. = 53.0 g), round (9.0 × 8.6 mm), red, and had a sweet-sour taste with slight bitterness. At the age of 12 years, the yield reached 2.3 kg per bush, with a fruiting score of 4.0. This form accumulated high levels of vitamin C (346.85 mg/%), vitamin B9 (193.24 mg/100 g), and vitamin B12 (11.52 mg/100 g), exceeding the average values by 1.87-, 1.74-, and 1.44-times, respectively. It also exhibited a high sugar content (9.6%) and dry matter content (17.7%), with an SAI of 3.47%.

Among the studied components of the bioflavonoid complex, rutin occupied a dominant position, with variations from 1.95 to 19.42 mg/100. The average value was 10.74 mg/100 g (Table 4).

The rutin content varied significantly, with a C% of 42.42%. The forms were divided into two classes: those with a high content (>9.69 mg/100 g) (five forms) and those with a low content (<9.68 mg/100 g) (eight forms). The highest values (15.8–19.4 mg/100 g) were recorded in the forms K (38-7), Barkhotinskaya (30-5), Zhemchuzhnoe Ozherel’e (32-8), Shtambovaya K (45-1), and K (46-1).

Robinin content ranged from 0.92 to 10.21 mg/100 g, with a variation coefficient of 41.34%. High values (>5.56 mg/100 g) were noted in six forms, with the maximum recorded in K (46-1) (10.21 mg/100 g). Apigenin was detected in small amounts in three forms, with the highest content observed in Nezhenka K (42-1) (0.24 mg/100 g). Hyperoside was found in one-third of the samples, with the highest accumulation recorded in K (28-6) (0.25 mg/100 g).

Gallic acid content ranged from 0.11 to 1.25 mg/100 g, with a variation coefficient of 41.0%. The highest values were found in Zhemchuzhnoe Ozhel’e (32-8) (1.25 mg/100 g) and K (46-1) (1.13 mg/100 g). Hypolaetin was detected in 10 out of 13 forms, with concentrations ranging from 0.14 to 0.47 mg/100 g, and the highest accumulation was observed in Luchistaya K (28-6) (0.47 mg/100 g).

The best varieties in terms of flavonoid composition included Zhemchuzhnoe Ozhel’e (32-8) (containing five flavonoid compounds), as well as Nezhenka (42-1), K (28-6), and K (46-1) (each containing three compounds).

The Zhemchuzhnoe Ozhel’e (32-8) form exhibited a bush height of 1.8 m, with a stem diameter of 2.4–2.8 cm, and demonstrated high winter hardiness. The brush size measured 5.07 × 5.71 cm, with an average of 32.8 fruits per brush. The fruits were medium-sized (100 pcs. = 55.9 g), round (8.2 × 8.0 mm), red, and had a sweet-sour taste with bitterness. At 12 years of age, the yield reached 2.8 kg per bush, with a fruiting score of 4.0. The bioflavonoid complex was dominated by rutin (18.48 mg/100 g) and robinin (9.87 mg/100 g), with smaller amounts of gallic acid (1.25 mg/100 g), hyperoside (0.23 mg/100 g), and hypolaetin (0.29 mg/100 g).

Based on the qualitative composition of flavonoids, six flavonoid compounds were identified in *V. opulus* forms. High rutin content was recorded in five forms: K (38-7), Barkhotinskaya (30-5), Zhemchuzhnoe Ozhel’e (32-8), Shtambovaya (45-1), and K (46-1). High robinin content was observed in seven forms: Nezhenka K (42-1), K (39-1), Zhemchuzhnoe Ozhel’e (32-8), Shtambovaya (45-1), K (28-6), K (46-1), and K (37-2). High gallic acid accumulation was noted in three forms: Zhemchuzhnoe Ozhel’e (32-8), K (28-6), and K (46-1). High hypolaetin content was found in six forms: K (38-7), Nezhenka K (42-1), Luchistaya (28-6), K (39-1), K (46-8), and Zhemchuzhnoe Ozhel’e (32-8). High hyperoside content was detected in two forms: Zhemchuzhnoe Ozhel’e (32-8) and K (28-6). High apigenin content was found in one form: Nezhenka K (42-1).

### 3.4. Biochemical Composition of Varieties and Forms of L. caerulea *subsp*. altaica

The collection of *L. caerulea* subsp. *altaica* in the ABS includes 22 forms, 105 seedlings of the elite forms Golubaya Volna and No. 2, and 26 clones of Golubaya Volna. For the analysis of BAS, 16 forms were studied, while the flavonoid composition was examined in 10 forms. The vitamin C content ranged from 35.52 mg/% (No. 23) to 55.00 mg/% (No. 5) (Table 5).

The accumulation of vitamin C in the fruits of *L. caerulea* subsp. *altaica* depended on the form. The maximum content (above 48.91 mg/%) was observed in forms No. 1, No. 5, No. 9, No. 17, and No. 18. The minimum values (35.52–41.98 mg/%) were recorded in forms No. 2, No. 4, No. 6, and No. 21–23. The average value was 42.76 mg/%, with a variation coefficient of 26.89%.

The concentration of sugars ranged from 6.8% to 9.7% (average—8.54%, variation coefficient—11.18%). High values (above 8.26%) were characteristic of forms No. 3–9, No. 17, No. 18, and No. 21. Forms No. 3, No. 5, No. 9, No. 17, and No. 18 exhibited a simultaneous increase in both sugar and vitamin C content. Organic acids (1.87–3.80%, average—2.75%) influenced the taste. The SAI fluctuated between 1.78% and 5.22% (average—3.28%), with the highest values recorded for forms No. 3, No. 4, and No. 6.

The dry matter content ranged from 9.8% to 17.6% (average—14.37%, variation coefficient—10.72%). The dry matter content exceeded 13.8% in 10 out of 16 forms. Forms No. 3–9 were distinguished by high sugar, acid, and dry matter content. Phenolic compounds varied from 739 mg/% (No. 4) to 820 mg/% (No. 19), with high values recorded for forms No. 1, No. 2, No. 5–9, No. 18, No. 19, No. 21, No. 23, and Golubaya Volna.

Based on biochemical parameters, the best forms were identified as No. 5, No. 7, and No. 9 (excelling in five parameters); No. 1, No. 6, No. 8, and No. 19 (in four parameters); and No. 3, No. 4, No. 17, No. 18, and No. 21 (in three parameters).

The analysis of flavonoid content indicated that hyperoside, isorhamnetin, and myricetin were present in all *L. caerulea* subsp. *altaica* samples, with average values of 76.83 mg/100 g, 5.79 mg/100 g, and 3.08 mg/100 g, respectively (Table 6).

Kaempferol was detected in eight forms, ranging from 9.13 to 51.25 mg/100 g (average—26.95 mg/100 g, variation coefficient—35.67%). Hypolaetin was identified in six forms (51.45–88.98 mg/100 g, average—66.54 mg/100 g, variation coefficient—19.26%), with the highest values observed in forms No. 9 and No. 10.

Rutin was found in three forms, with significant concentrations in No. 7 (28.60 mg/100 g) and No. 10 (14.30 mg/100 g), while only a minimal amount was detected in No. 2 (1.66 mg/100 g). It was absent in seven forms. Luteolin was identified in forms No. 1, No. 5, and No. 7, ranging from 3.42 to 4.01 mg/100 g (average—3.68 mg/100 g).

Gallic acid was isolated from two forms (No. 7 and No. 8) in amounts ranging from 29.04 to 36.31 mg/100 g, while it was not detected in the other forms. Myricetin was present in all forms, with concentrations ranging from 0.11 to 9.66 mg/100 g; the highest content was recorded in forms No. 4 (9.65 mg/100 g) and No. 7 (5.73 mg/100 g).

Hyperoside was detected in all forms, varying from 28.78 to 129.99 mg/100 g (average—76.83 mg/100 g, variation coefficient—38.70%), with the highest content found in forms No. 7, No. 8, and No. 9. Isorhamnetin was also present in all forms (1.70–9.41 mg/100 g, average—5.79 mg/100 g), with maximum values recorded in forms No. 4, No. 5, No. 6, No. 7, and No. 8. Robinin was not detected in the fruits of *L. caerulea* subsp. *altaica*.

**Figure 4:**
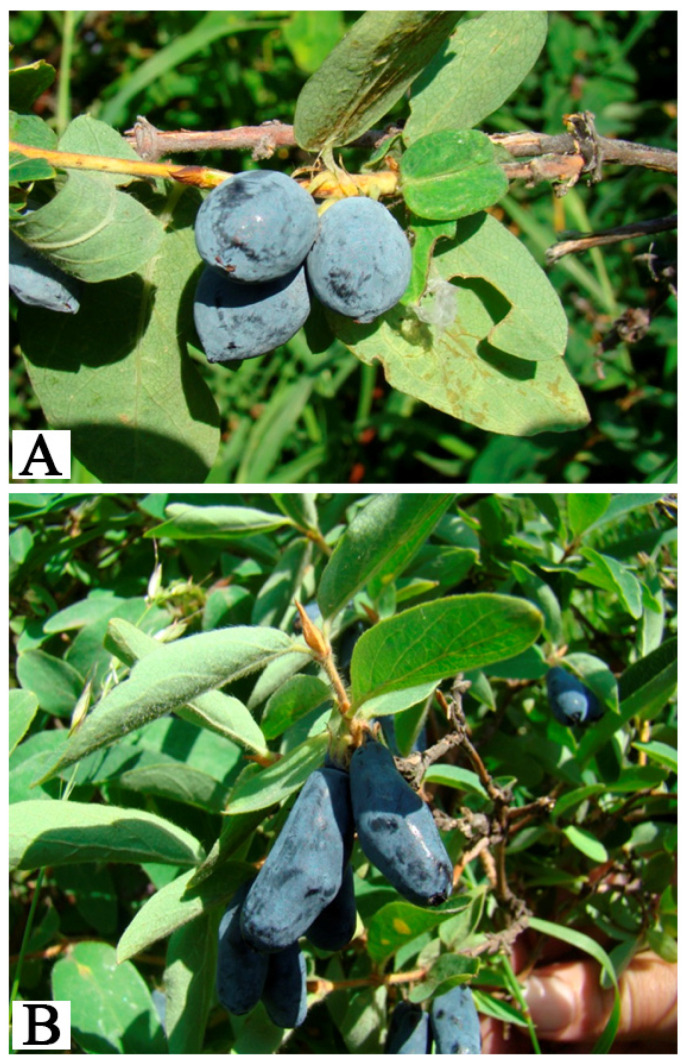
Fruits of *L. caerulea* subsp. *altaica*. (**A**)—Form No. 4; (**B**)—Form No. 7.

Form No. 4 was classified as mid-season, with a bush height of 1.5 m. The fruits were oval-elongated (13.5 × 9.0 mm, index 1.5), weighed 0.66 g, and had a sour taste with bitterness. Productivity reached 1.9 kg per bush. This form was distinguished by a high content of vitamin C (54.8 mg/%) and phenolic compounds (780 mg/kg), along with significant accumulation of hyperoside, hypolaetin, kaempferol, myricetin, and isorhamnetin (Figure 4A).

Form No. 7 was classified as late-ripening, with a bush height of 1.2 m. The fruits were spindle-shaped (18.8 × 8.8 mm, index 2.1), weighed 0.56 g, and had a sweet and sour taste. Productivity reached 2.5 kg per bush. The fruits were rich in dry matter (10.0%) and phenolic compounds (775 mg/g) and were distinguished by a high content of hyperoside, gallic acid, rutin, kaempferol, isorhamnetin, and myricetin (Figure 4B). The maximum content of individual compounds was recorded as follows: myricetin—No. 4 (9.65 mg/100 g) and No. 7 (5.73 mg/100 g); hypolaetin—No. 9 (88.98 mg/100 g) and No. 10 (77.85 mg/100 g); rutin—No. 7 (28.60 mg/100 g); kaempferol—No. 4 (46.25 mg/100 g), No. 9 (48.32 mg/100 g), and No. 10 (51.25 mg/100 g); isorhamnetin—No. 4 (7.21 mg/100 g), No. 5 (9.41 mg/100 g), No. 6 (7.31 mg/100 g), No. 7 (7.51 mg/100 g), and No. 8 (7.0 mg/100 g); luteolin—No. 7 (4.00 mg/100 g); gallic acid—No. 8 (36.31 mg/100 g); hyperoside—No. 4 (73.33 mg/100 g), No. 7 (116.99 mg/100 g), No. 8 (129.99 mg/100 g), and No. 9 (110.14 mg/100 g).

In terms of flavonoid composition, form No. 7 contained the highest number of compounds (seven), followed by No. 8 and No. 10 (six compounds each), and No. 2, No. 4, No. 5, No. 6, and No. 9 (five compounds each).

### 3.5. Comparison of the Biochemical Composition of Fruits Among Three Species

To assess the genetic determinacy of the traits, the coefficients of variation were used to reflect their dependence on the genotype. A comparison of the accumulation levels and variability of biochemical characteristics showed that most of the analyzed traits exhibited high or very high variability. In particular, the vitamin C content in fruits varied with a coefficient of variation ranging from 26.89% to 45.41% (Table 7).

The highest accumulation of vitamin C was observed in *V. opulus* (185.42 mg/%; C%—33.44%), followed by *H. rhamnoides* (66.44 mg/%; C%—45.41%) and *L. caerulea* subsp. *altaica* (42.76 mg/%; C%—26.89%). The SAI varied at a high level in *H. rhamnoides* (C%—35.24%) and *L. caerulea* subsp. *altaica* (C%—26.64%). *H. rhamnoides* also exhibited high variability in phenolic compounds (C%—28.67%) and carotenoids (C%—38.71%), whereas *V. opulus* showed high variability in robinin, rutin, and gallic acid (C%—41.34–44.37%). In *L. caerulea* subsp. *altaica*, significant variability was noted for hyperoside (C%—38.70%), myricetin (C%—45.63%), kaempferol (C%—35.67%), and isorhamnetin (C%—24.51%). An average level of variability was observed for titratable acidity (C%—13.48–21.71%) and hypolaetin (C%—19.26%). The most stable indicators were the total sugar content (C%—8.71–11.18%) and dry matter (C% < 12%).

A comparison of the biochemical composition showed that *H. rhamnoides* exhibited the greatest variability (54.5%), followed by *L. caerulea* subsp. *altaica* (36.4%) and *V. opulus* (22.2%). Robinin (4.13 mg/100 g) and rutin (2.77 mg/100 g) were detected in *H. rhamnoides*, along with trace amounts of gallic acid, hypolaetin, and hyperoside. In *V. opulus*, robinin (6.04 mg/100 g), rutin (10.74 mg/100 g), and apigenin (0.15 mg/100 g) were identified, along with minor concentrations of gallic acid, hypolaetin, and hyperoside. *L. caerulea* subsp. *altaica* contained eight flavonoids, including high levels of gallic acid (32.67 mg/100 g), hypolaetin (66.8 mg/100 g), kaempferol (29.51 mg/100 g), and rutin (14.85 mg/100 g), which were significantly higher than those found in *H. rhamnoides* and *V. opulus*.

## 4. Discussion

The biochemical composition of the fruits of *H. rhamnoides*, *V. opulus* and *L. caerulea* subsp. *altaica* was analyzed to identify promising forms with elevated levels of bioactive compounds. The content of chemical substances was examined in *H. rhamnoides* for 12 parameters and in *V. opulus* and *L. caerulea* subsp. *altaica* for 14 parameters.

The results demonstrated that the fruits of the studied species accumulated a diverse range of beneficial compounds, including vitamin C, sugars, phenolic substances, and flavonoids. This composition makes them valuable for various practical applications, including their use as a genetic resource for breeding programs aimed at enhancing fruit biochemical composition.

Significant genotypic differences were identified in the accumulation patterns of individual compounds, indicating varying degrees of genetic determination. This differentiation enabled the identification of traits with distinct levels of variability, which can be utilized for targeted modification of fruit biochemical composition through selective breeding. A comparative analysis of the coefficients of variation for more than 18 biochemical traits in the fruits of *H. rhamnoides*, *V. opulus*, and *L. caerulea* subsp. *altaica* (Table 7) revealed a broad range of variation. This allowed for the identification of traits with the highest and lowest levels of genotype dependency. *H. rhamnoides* exhibited the greatest variability across several parameters. The majority of the biochemical traits in *L. caerulea* subsp. *altaica* also displayed a high level of variability with 63.6% of traits showing variability > 20%. In *V. opulus*, traits were distributed almost equally among three levels of variability—low, medium, and high—each accounting for approximately C% = 22%.

To improve the organoleptic properties of the studied fruits, special attention was given to identifying varieties and forms with reduced titratable acid content while maintaining high soluble sugar levels, in accordance with previous studies [39,40].

Biochemical studies on fruit composition have been conducted in multiple countries. A comparison of *H. rhamnoides* data from different regions indicated no significant differences in the accumulation of chemical substances. Specifically, vitamin C levels in this species varied widely. Populations from the Altai Mountains, one of the most studied regions in Siberia, exhibited vitamin C concentrations ranging from 50–100 mg/%, with some exceeding 200 mg/%. High vitamin C content was also reported in *H. rhamnoides* from the Western Pamirs and Central Asia [41]. Conversely, plants from the North Caucasus contained lower levels of vitamin C, ranging from 18.7 to 86.6 mg/% [42]. This variation suggests that vitamin C accumulation in *H. rhamnoides* fruits is primarily influenced by genotype and variety. According to the literature, the vitamin C content of *H. rhamnoides* fruits ranges from 52.86 to 896 mg/100 g [43], which mean that forms Pamyati Baytulina (156 mg/%) and Dolgozhdannaya No. 5 (3-24) III (146.64 mg/%) (Table 1) could be considered promising.

The carotenoid content of promising *H. rhamnoides* forms and varieties was comparable to β-carotene-rich Altai cultivars and wild populations from Central Asia and the Kaliningrad region [44,45]. For example, studies have reported total carotenoid contents ranging from 53 to 97 mg/100 g of dry weight in berries from six Romanian *H. rhamnoides* varieties [46], which was higher that the values found in our study.

The accumulation patterns of sugars, dry matter, and acidity were consistent with findings from other countries. In *V. opulus*, chemical composition data showed greater variation than reported by other researchers. The studied plants exhibited a high vitamin C content, reaching 346 mg/%, which was 1.5 to 2 times higher than levels found in plants from Canada and Russia [23,47,48,49]. This suggests that the natural ecological niche of the studied populations was optimal for vitamin C accumulation. Additionally, the dry matter content was observed to be as high as 18%. Researchers have also investigated the fractional composition and total content of lipids in these species [20,50].

In *L. caerulea* subsp. *altaica*, the strongest genotypic dependence was noted in the accumulation patterns of all components of the bioflavonoid complex and vitamin C. These results align with previous findings [51].

Special attention should be given to varieties and forms with a high content of BAS and flavonoids. In *H. rhamnoides*, promising genotypes include Pamyati Baitulina, Fakel K-14-81 (3-25), and the forms Solnyshko (1-18) and Dolgozhdannaya No. 5 (3-24). For *V. opulus*, the notable forms are Shtambovaya (45-1) and Zhemchuzhnoe Ozhel’e (32-8), while *L. caerulea* subsp. *altaica* includes forms No. 4, No. 5, and No. 7.

In *H. rhamnoides*, moderate variations were observed in the accumulation of vitamin C (C%—45.40%), SAI (C%—35.24%), carotenoids (C%—38.71%), phenolic substances (C%—28.67%), robinin (C%—32.12%), rutin (C%—30.96%), and gallic acid (C%—41.03%), suggesting varying degrees of genetic determination. In *V. opulus*, high variability was detected for vitamin C (C%—33.44%), robinin (C%—41.34%), rutin (C%—42.42%), gallic acid (C%—41.00%), and hypolaetin (C%—29.70%). In *L. caerulea* subsp. *altaica*, the coefficient of variation for vitamin C was 26.89%, for SAI—26.64%, and for flavonoids: hyperoside (C%—38.70%), myricetin (C%—45.63%), and kaempferol (C%—35.66%).

The absence of significant interspecific differences in sugar, acidity, and dry matter content suggests a general biological mechanism underlying the accumulation of these substances. Similar patterns have been reported in previous studies [52,53,54]. In *V. opulus* and *L. caerulea* subsp. *altaica*, the accumulation of bioflavonoids and vitamin C was most strongly influenced by genotype. In *H. rhamnoides*, phenolic substances and carotenoids also exhibited strong genetic determination.

Although the study focused on specific populations of *H. rhamnoides*, *V. opulus*, and *L. caerulea* subsp. *altaica*, which may limit the generalizability of the results to other regions with different environmental conditions, the high content of bioactive compounds in the studied fruits suggests their potential for use in functional food products and nutraceuticals. The significant accumulation of vitamin C in *H. rhamnoides*, with concentrations comparable to or exceeding those found in other global populations, supports its role as a potent natural antioxidant. Additionally, the presence of carotenoids, phenolic compounds, and flavonoids enhances the fruits’ health-promoting properties, including anti-inflammatory, cardioprotective, and immune-boosting effects. The promising genotypes identified in this study, such as *H. rhamnoides* Pamyati Baitulina and Dolgozhdannaya No. 5, *V. opulus* Shtambovaya and Zhemchuzhnoe Ozherel’e, and *L. caerulea* subsp. *altaica* forms No. 4, No. 5, and No. 7, can serve as valuable resources for such breeding initiatives. These findings can facilitate the development of health-focused food products and dietary supplements enriched with bioactive compounds.

## 5. Conclusions

The biochemical composition of *H. rhamnoides*, *V. opulus*, and *L. caerulea* subsp. *altaica* exhibits substantial genotypic variability, particularly in the accumulation of vitamin C, flavonoids, phenolic substances, and carotenoids. This diversity highlights the potential of these species as valuable genetic resources for breeding programs aimed at improving fruit quality and nutritional value. While sugar, acidity, and dry matter content show minimal interspecific differences, the accumulation of bioactive compounds is strongly influenced by genotype, especially in *V. opulus* and *L. caerulea* subsp. *altaica*. Several promising varieties and forms with high levels of bioactive substances were identified, providing a foundation for future selection and genetic enhancement of these fruit-bearing species. Notable genotypes include *H. rhamnoides* (Pamyati Baitulina, Fakel K-14-81 (3-25), Solnyshko (1-18), Dolgozhdannaya No. 5 (3-24)); *V. opulus* (Shtambovaya (45-1), Zhemchuzhnoe Ozherel’e (32-8)); and *L. caerulea* subsp. *altaica* (Forms No. 4, No. 5, No. 7). These findings have important practical implications, particularly for the food and pharmaceutical industries. The identified genotypes with high concentrations of bioactive compounds could be utilized for the development of functional foods, dietary supplements, and natural antioxidants. Moreover, their potential use in breeding programs can contribute to the production of improved cultivars with enhanced health benefits, better adaptability to environmental conditions, and superior organoleptic properties.

## Figures and Tables

**Table 1:** Biochemical parameters of *H. rhamnoides* fruits.

Variety, Form	Vitamin C, mg/%	Total Sugar, %	Titratable Acidity, %	Sugar–Acid Index (SAI)	Dry Matter, %	Carotenoids, mg/100 g	Phenolic Compounds, mg/100 g
Vitaminka T-14-86 (3-20) III	26.00	4.61	1.35	3.41	10.0	39.13	63.6
Roskosh (3-5) III	52.04	3.68	2.38	1.54	9.8	25.0	51.0
Krasnoplodnaya K-14-81 (4-27) III	38.16	3.56	2.79	1.27	8.4	55.30	52.8
Plakuchaya T-2-82 (2-32) III	41.60	3.54	2.02	1.75	8.2	20.38	51.0
Kan 2-86 (2-4) II	34.32	4.20	1.74	2.41	8.6	16.62	76.5
Fakel K-14-81 (3-17) III	57.20	4.13	1.67	2.47	9.2	31.43	59.0
Dolgozhdannaya No. 5 (3-24) III	146.64	3.96	1.85	2.14	10.0	8.40	92.0
Feyerverk K-14-81 (3-4) II	26.00	4.08	1.65	2.47	10.0	19.57	58.3
Lyubimaya T-2-82 (3-14) II	60.50	3.62	2.58	1.40	10.0	15.80	87.7
Pamyati Baytulina	156.00	3.68	1.94	1.89	10.4	25.04	105.8
Sh-9-81 (4-6) II	23.40	3.54	2.56	1.38	9.4	13.39	52.2
Yuubileinaya Kotuhova T-2-82 (2-22) III	140.92	3.45	2.86	1.20	10.2	21.70	100.46
Solnyshko (1-18) III	120.38	4.35	1.63	2.58	10.0	8.39	77.5
Shetlastinka No. 7 (2-24) III	33.80	3.64	1.95	1.78	10.5	24.17	53.3
Yantarnaya (2-1) III	31.20	3.51	2.36	1.75	10.4	23.11	41.9
Asem Sh-9-81 (3-27) III	78.00	3.72	2.13	1.74	11.8	22.30	71.5
Mean value	66.64 ± 24.60	3.83 ± 0.18	2.09 ± 0.24	1.95 ± 0.69	9.8 ± 0.47	23.11 ± 6.06	68.41 ± 10.25

**Table 2:** Flavonoid composition of fruits of *H. rhamnoides* varieties and promising forms.

Variety, Form	Flavonoid Content, mg/100 g				
	Robinin	Rutin	Gallic Acid	Hypolaetin	Hyperoside
Yubileinaya Kotuhova T-2-82 (2-22) III	3.612	1.191	0.218	0	0
Dolgozhdannaya No. 5 (3-24) III	4.809	1.637	0.726	0.189	1.284
Krasnoplodnaya K-14-81 (4-27) III	2.719	3.694	0.411	0	0
Vitaminka T-14-86 (3-20) III	1.314	2.965	0.229	0	0
Asem Sh-9-81	7.524	2.589	0.363	0	0
(3-27) III	4.721	1.878	0.242	0	0
Shetlastinka No. 7 (2-24) III	4.524	4.767	0.291	0	0
Mean value	4.17 ± 1.54	2.67 ± 0.99	0.35 ± 0.14	–	–

**Table 3:** Biochemical parameters of fruits of *V. opulus* promising forms.

Form	Vitamin C, mg/%	Total Sugar, %	Titratable Acidity, %	Sugar–Acid Index (SAI)	Dry Matter, %	Vitamin B9, mg/100 g	Vitamin B2, mg/100 g	Vitamin B12, mg/100 g
Blestyashhaya (45-7)	125.36	9.4	1.88	5.00	17.2	122.48	0.15	0.41
Shtambovaya (45-1)	346.85	9.6	2.77	3.47	17.7	193.24	1.13	11.52
K (34-5)	205.31	8.1	2.46	3.29	15.1	84.32	0.26	7.77
K (31-4)	116.18	7.7	2.21	3.48	14.4	75.72	1.43	8.44
Luchistaya (28-6)	211.53	7.8	2.04	3.82	14.4	110.07	1.29	6.89
K (27-4)	209.64	8.7	1.89	4.60	16.1	118.00	1.00	6.00
Nezhenka (42-1)	137.87	9.6	2.17	4.42	18.0	120.94	1.60	5.90
K (45-2)	145.85	7.7	2.14	3.60	14.3	64.96	1.68	5.06
Businka (24-7)	139.40	8.1	2.43	3.33	15.0	88.50	1.15	10.72
K (28-2)	209.14	8.2	2.16	3.80	15.2	73.96	1.20	9.84
K (22-5)	215.17	7.4	1.87	3.96	13.7	114.50	1.30	9.00
K (23-6)	127.90	8.7	1.99	4.37	16.1	82.03	1.29	8.74
K (27-5)	140.87	7.7	1.76	4.38	13.9	116.78	1.37	6.90
K (23-8)	146.68	8.7	2.23	3.90	16.1	105.41	0.99	8.43
K (35-4)	243.47	7.9	2.34	3.38	14.5	194.16	1.26	6.75
Mean value	185.42 ± 34.67	8.35 ± 0.39	2.16 ± 0.15	3.92 ± 0.28	15.45 ± 0.74	111.00 ± 20.82	1.21 ± 0.19	7.99 ± 1.05

**Table 4:** Flavonoid content in fruits of *V. opulus* promising forms.

Form	Flavonoid Content, mg/100 g					
	Hypolaetin	Rutin	Apigenin	Hyperoside	Gallic Acid	Robinin
K (31-6)	0	5.244	0	0	0.183	0.918
K (38-7)	0.369	19.421	0	0	0.191	5.429
Nezhenka (42-1)	0.441	5.326	0.246	0.075	0.241	6.334
Luchistaya (28-6)	0.476	8.581	0	0	0	3.393
K (39-1)	0.357	7.124	0	0	0.249	7.854
(30-5)	0.226	19.321	0	0.143	0.419	4.171
(46-8)	0.334	8.374	0	0	0.266	2.443
Dyhanie oseni (23-6)	0.227	4.298	0	0	0.182	4.124
Zhemchuzhnoe Ozherel’e (32-8)	0.252	18.478	0	0.225	1.254	9.874
Shtambovaya (45-1)	0.334	15.852	0	0	0.508	8.524
K (28-6)	0	1.957	0	0.251	0.652	5.421
K (46-1)	0.148	17.759	0.135	0	1.137	10.215
K (37-2)	0	7.985	0.087	0	0.119	9.867
Mean values *	0.316 ± 0.07	10.748 ± 3.72	0.156 ± 0.05	0.174 ± 0.06	0.450 ± 0.23	6.044 ± 1.76

*—mean values were calculated only for samples where this component is present.

**Table 5:** Biochemical analysis of fruits of *L. caerulea* subsp. *altaica*.

Form	Vitamin C, mg/%	Total Sugar, %	Titratable Acidity, %	Sugar–Acid Index (SAI)	Dry Matter, %	Phenolic Compounds, mg/100 g
No. 17	53.12	8.5	2.46	3.45	11.6	750
No. 18	54.80	9.4	2.42	3.88	12.8	780
No. 19	45.58	7.2	3.20	2.25	15.8	820
No. 21	40.18	9.3	3.61	2.57	9.8	805
No. 22	38.14	7.8	3.31	2.35	10.0	775
No. 23	35.52	8.1	3.49	2.32	11.6	790
Golubaya Volna	44.09	6.8	3.80	1.78	13.2	788
No. 1	52.30	7.8	2.56	3.04	14.5	815
No. 2	41.98	7.6	2.41	3.15	14.3	801
No. 3	46.72	8.2	1.87	5.22	15.6	736
No. 4	38.49	9.3	2.33	3.99	17.0	739
No. 5	55.00	9.6	2.55	3.76	17.0	796
No. 6	39.90	9.4	2.32	4.05	17.1	791
No. 7	44.53	9.7	2.70	3.59	16.9	820
No. 8	43.27	9.7	2.58	3.75	17.6	792
No. 9	50.93	8.2	2.41	3.40	15.2	789
Mean values	42.76 ± 3.29	8.54 ± 0.50	2.75 ± 0.29	3.28 ± 0.46	14.37 ± 1.36	786.69 ± 13.56

**Table 6:** Flavonoid composition of fruits of *L. caerulea* subsp. *altaica*.

Form	Flavonoid Content, mg/100 g							
	Myricetin	Hypoelastin	Rutin	Kaempferol	Isoramenthin	Luteolin	Gallic Acid	Hyperoside
No. 1	2.585	0	0	0	4.709	3.428	0	61.499
No. 2	0.924	0	1.668	15.217	4.108	0	0	28.782
No. 3	1.664	0	0	0	4.591	0	0	56.638
No. 4	9.659	51.456	0	46.256	7.214	0	0	73.335
No. 5	0.112	64.974	0	21.325	9.417	3.654	0	0
No. 6	1.109	59.322	0	9.131	7.312	0	0	49.211
No. 7	5.734	0	28.605	13.266	7.515	4.005	29.048	116.995
No. 8	3.699	58.231	0	10.896	7.012	0	36.311	129.991
No. 9	2.778	88.983	0	48.321	4.308	0	0	110.141
Golubaya Volna	2.514	77.854	14.302	51.254	1.703	0	0	64.995
Mean value *	3.000 ± 1.85	66.800 ± 10.11	14.850 ± 8.69	26.950 ± 15.11	5.740 ± 1.49	3.680 ± 0.19	32.67 ± 2.34	76.830 ± 21.38

*—mean values were calculated only for samples where this component is present.

**Table 7:** Biochemical composition of fruits of *H. rhamnoides*, *V. opulus*, *L. caerulea* subsp. *altaica*.

Fruit Component	*H. rhamnoides*			*V. opulus*			*L. caerulea* subsp. *altaica*		
	M ± SD Min–Max	C%	P%	M ± SD Min–Max	C%	P%	M ± SD Min–Max	C%	P%
Vitamin C, mg/%	66.64 ± 24.60 23.40–156.00	45.40	9.21	185.42	33.44	8.9	42.76 ± 6.01 35.52–55.0	26.89	6.72
Total sugar, %	3.83 ± 0.18 3.51–4.61	9.07	2.27	8.35 ± 0.41 7.40–9.60	8.71	2.33	8.54 ± 0.50 6.8–9.7	11.18	2.79
Titratable acidity, %	2.09 ± 0.27 1.35–2.86	21.71	5.43	2.16 ± 0.15 1.76–2.77	13.48	3.34	2.75 ± 0.29 1.87–3.80	20.07	5.02
Sugar–acid index (SAI)	1.89 ± 0.35 1.20–3.41	35.24	8.81	3.92 ± 0.28 3.29–5.00	13.38	3.46	3.28 ± 0.46 1.78–4.05	26.64	6.66
Dry matter, %	9.81 ± 0.47 8.20–11.80	9.13	2.28	15.45 ± 0.76 13.7–18.00	8.85	2.37	14.37 ± 1.36 9.8–17.6	10.72	3.05
Phenolic compounds, mg/100 g	68.41 ± 10.25 41.09–105.80	28.67	7.17	–	–	-	786.69 ± 13.55 739–820	3.29	3.82
Carotenoids, mg/100 g	23.11 ± 6.06 8.39–55.30	38.71	8.40	–	–	-	-	-	-
Tocopherols, mg/100 g	2.48 ± 0.23 2.10–3.40	16.98	4.39	–	–	-	-	-	-
Robinin, mg/100 g	4.18 ± 1.54 1.31–7.52	32.12	9.84	6.04 ± 1.76 0.92–10.22	41.34	9.07	-	-	-
Rutin, mg/100 g	2.67 ± 0.99 1.19–4.77	30.96	10.20	10.74 ± 3.72 1.96–19.42	42.42	10.20	14.85 1.67–28.61	-	-
Gallic acid, mg/100 g	0.35 ± 0.14 0.21–0.72	41.03	10.3	0.45 ± 0.23 0.12–1.25	41.00	12.24	32.67 29.05–36.31	-	-
Hypolaethin, mg/100 g	0.189	–	–	0.32 ± 0.07 0.45–0.48	29.70	8.4	66.54 ± 10.12 51.46–88.98	19.26	7.28
Hyperoside, mg/100 g	1.284	–	–	0.174 0.08–0.25	–	-	76.83 ± 21.38 28.78–129.99	38.70	9.31
Apigenin, mg/100 g	–	–	–	0.156 0.09–0.25	–	-	-	-	-
Myricetin, mg/100 g	-	-	-	-	-	-	3.08 ± 1.85 0.11–9.66	45.63	28.79
Kaempferol, mg/100 g	-	-	-	-	-	-	29.51 ± 14.38 9.13–51.25	35.67	9.80
Isorhamnetin, mg/100 g	-	-	-	-	-	-	5.79 ± 1.49 1.70–9.42	24.51	7.09
Luteolin, mg/100 g	-	-	-	-	-	-	3.68 3.42–4.01	-	-
Quercetin, mg/100 g	-	-	-	-	-	-	-	-	-

M ± SD—mean value ± standard deviation; C%—variation coefficients; P%—experimental accuracy.

## Data Availability

All the data mentioned in the article are included in the main body of the manuscript.

## Abbreviations

The following abbreviations are used in this manuscript:

ABG	Altai Botanical Garden
BAS	Biologically active substances
HPLC	High-performance liquid chromatography
SAI	Sugar–Acid index
TLC	Thin-layer chromatography
